# Isolation and characterization of *Escherichia coli* pathotypes and factors associated with well and boreholes water contamination in Mombasa County

**DOI:** 10.11604/pamj.2016.23.12.7755

**Published:** 2016-01-22

**Authors:** Thani Suleiman Thani, Samwel Morris Lifumo Symekher, Hamadi Boga, Joseph Oundo

**Affiliations:** 1 Field Epidemiology Training Program, Kenya; 2 Kenya Medical Research Institute, Nairobi, Kenya; 3 Jomo Kenyatta University of Agriculture and Technology, Nairobi, Kenya; 4 Walter Reed Program, kenya

**Keywords:** Wells, Bore holes, Coliforms, Escherichia coli, antibiotic susceptibility

## Abstract

**Introduction:**

Safe water for human consumption is important, but there is a limited supply. Mombasa County has water shortages making residences rely on other sources of water including boreholes and wells. Microbiological evaluation of drinking water is important to reduce exposure to water borne enteric diseases. This cross sectional study aimed at determining the frequency and characterization of *Escherichia coli (E. coli)* pathotypes from water samples collected from wells and boreholes in Mombasa County.

**Methods:**

One hundred and fifty seven (157) water samples were collected from four divisions of the county and a questionnaire administered. The samples were inoculated to double strength MacConkey broth and incubated at 370C for up to 48 hours. Positive results were compared to the 3 tube McCrady MPN table. The *E. coli* were confirmed by Eijkman's test and antibiotic susceptibility carried out. Using polymerase chain reaction (PCR), the *E. coli* were characterized to establish pathotypes.

**Results:**

One hundred and thirty one (n = 131; 83.4%) samples had coliform bacteria with only 79 (60.3%) samples having *E. coli*. Significant values (<0.05) were noted when coliforms were compared to variables with *E. Coli* showing no significance when compared to similar variables. *E. coli* (n = 77; 100%) tested were sensitive to Gentamicin, while all (n = 77; 100%) isolates were resistant to Ampicillin. PCR typed isolates as enteroinvasive *E. Coli (EIEC)*.

**Conclusion:**

Findings suggest that coliforms and *E. coli* are major contaminants of wells and boreholes in Mombasa County. The isolates have a variety of resistant and sensitivity patterns to commonly used antibiotics.

## Introduction

Water is an important resource that is prone to bacterial contamination from a variety of hosts including mammals and avian species [1]. The rapid expansion of Mombasa County has led to the residents relying on groundwater to supply them with portable water [2]. Drinking water comes from surface water and ground water. Surface water includes rivers, lakes, and reservoirs while Ground water is pumped from wells or boreholes that are drilled into aquifers. However due to dwindling resources and faulty sanitation especially in developing world makes the availability of safe water almost unattainable, this is due to bacterial and chemical contamination [2]. Water-borne diseases are one of the major public health problems in developing countries. It is estimated that contaminated water has caused more than 20 million deaths [1], of which more than 80% were among children under age five [3]. In the developing world, more than one billion people have no safe drinking water, or water for washing their food, hands and utensils before eating, while 2.4 billion also have no adequate sanitation [4]. This leads to; water-borne diseases (e.g. cholera, typhoid), water-related diseases (e.g. malaria, yellow fever, river blindness, sleeping sickness), water-based diseases (e.g. guinea worm and bilharzias), water-scarce diseases (trachoma and scabies), diarrhea. Mombasa and the Coast province experience perennial water shortages. There is no sewerage system except within Mombasa Island. Shallow wells are dug near toilets or septic pits. Outbreaks of cholera and dysentery occur during raining seasons or shortly after the rains [2, 5]. Exchange of microbes between wells and toilets/septic pits has been documented [6]. Mombasa gets most of its water from Mzima springs, Marere and Baricho water works. However ground water forms an important source of water for Mombasa and its environs. This study intends to isolate and characterize *Escherichia coli* pathotypes and possible factors associated with wells and boreholes water contamination in Mombasa County [2].

## Methods

This cross sectional study was conducted in Mombasa County in the coastal region of Kenya. Mombasa County has a tropical type of climate with wet and dry climate. The County is divided into 4 divisions: Mainland North (Kisauni), Mainland South (Likoni), Island (Mvita) and Mainland West (Changamwe). A probability proportional cluster sampling method was used to provide the best estimate of the number of samples to be collected from each division in Mombasa County. A list of all the bore hole and water well in all the four divisions was obtained from the Public Health Office at Mombasa Public Health Department. To decide on the number of well and borehole to be sampled from each division the following formulae was used.

n = (required samples size) X (total number of well per division)/(Total number of well and bore well in the county)

After getting the number of wells/bore holes to be sampled per division, wells and borehole were assigned numbers and simple random sampling was used to identify the well and boreholes to be sampled. Water samples (200mls) were collected from bore holes and wells using sterile water collection labeled bottles. For water that had been treated with chlorine, 5% sodium thiosulfate was added to the sterile bottles to neutralize the chlorine. The bottles were placed in a cool box and transported to the laboratory for processing. At the site of collection, a questionnaire was administered to assess and determine the risk factors.

### Laboratory procedures

**Coliform contamination:** in order to determine coliform contamination, from the 200 ml water sample, 3 bottles each with 10ml of double strength MacConkey broth were inoculated with 10ml of water, 3 bottles each with 5ml of single strength Mac Conkey broth were inoculated with 1ml of water and another 3 bottles each with 5ml of single strength Mac Conkey broth were inoculated with 0.1ml of water. The bottles were then incubated at 37° C for up to 48 hours. The bottles were checked for lactose fermentation (yellow coloration) and gas production (air bubble in Durham tube). The number of positive samples was compared with 3- tube MPN McCradys Table to determine the most probable number of coliforms in the contaminated samples.

**Eijkman's test to detect faecal *E. coli*:** all positive bottles from the previous tests were sub-cultured into fresh single strength MacConkey broth and peptone water and incubated at 44.5 + 0.2°C for 48 hours. After incubation the MacConkey bottles were checked for lactose fermentation (yellow coloration) and gas production (bubble in Durham tube). All positive MacConkey bottles had Kovacs reagent added in the corresponding peptone water to detect indole production (red coloured ring). Those found to be positive were noted as positive for faecal *E. coli*.

**Antibiotic sensitivity testing:** this was determined by picking1-2 colonies of the isolated organism to obtain 0.5 MacFarland standard, then was spread/streaked onto the Muller Hinton agar and the disc for drugs (Table) were be placed on the media. The plates were incubated at 35°C for 18-24 hours and the zones of inhibition measured. The isolates were categorized as resistant, intermediate and sensitive to each antimicrobial agent as recommended by the National Committee for Clinical Laboratory Standards.

**DNA extraction and polymerase chain reaction:** the positive *E. coli* were inoculated to trypticase soy broth and incubated at 37°C for 16 to 18 hours. The DNA was extracted using the QIAGen DNA extraction procedure as per the kit manufacturer's instructions. Two molecular characterization tests were carried out to subtype the *E. coli* bacteria isolated from the study. First, a conventional PCR was carried out using CDC primers for subtyping *E. coli* subtypes EPEC, ETEC and EAEC. A master mix with the following was constituted, 2.5µl of 10× PCR buffer (Invitrogen, USA), 2.5µl of magnesium chloride (MgCl2) (Invitrogen, USA), 2µl of 0.4mM dNTPs (Invitrogen, USA), 0.4µl of each of the primers (forward and reverse primers) 7.75µl nuclease free water and 0.25µl of Taq polymerase (Qiagen, USA). A 3µl aliquot of DNA was added to give a final volume of 20µl. The cycling conditions for the PCRs were: incubation at 96°C for 4 minutes to activate the Taq polymerase. This was followed by 35 cycles involving denaturation at 95°C for 30 seconds, annealing at 57°C for 30 seconds and strand extension at 720C for 1 minute. Finally, a final incubation at 72°C for 10 minutes followed to fill in the recessed ends of the amplification products. This was carried out on a GeneAmp 9700 (Applied Biosystems, USA). The PCR products were visualized under UV light after gel electrophoresis using Tris-borate EDTA (TBE) buffer on 2% agarose gels stained with ethidium bromide at 100V for 60 minutes. The second molecular characterization tests was a real time PCR carried out to subtype all *E. coli* subtypes (EHEC, ETEC, EPEC, EAEC, EIEC and DAEC) after reconstitution of the following master mix; 10µl of QuantiTect PCR Probe (Qiagen, USA) Master mix buffer, 0.5µl of each of the primers (forward and reverse primers) and probe, 5.5µl nuclease free water and 3µl of DNA template was added to give a final volume of 20µl. The cycling conditions for the PCRs were: incubation at 95°C for 15 minutes to activate the Taq polymerase. This was followed by 45 cycles involving denaturation at 95°C for 15 seconds, annealing and extension at 55°C for 60 seconds. This was carried out on a RotorGene Q (Qiagen, USA).

## Results

### Description of sampling sites, samples

A total of 157 samples were collected around Mombasa County from the following areas of Likoni (n= 51; 32.5%), Island (n= 39; 24.8%), Changamwe (n= 37; 23.6%) and Kisauni (n= 30; 19.1%). During the study, majority of the samples were collected from boreholes (n= 98; 62.4%), while the other samples were collected from wells (n= 59; 37.6%). Majority of the water sources samples were protected (n= 144; 91.7%), while a few of the sampled water sources were not protected (n= 13; 8.3%). Many of water sources sampled had pumps available (n= 145; 92.4%) at the water source compared to a few that had no water pump (n= 12; 7.6%) available at the site. Majority of water sources samples that had pumps available at the water source had not recently over hauled/repaired (n= 137; 87.3%) compared to a few that had the water pump repaired/overhauled (n= 20; 12.7%). Of the 157 samples collected from water sources around Mombasa County, only 91 representing 58% were treated by the use of chlorine. The remaining 66 representing 42% were not treated with chlorine.

### Coliform contamination

Out of the 157 samples collected from water sources around Mombasa County and inoculated to Mac Conkey Broth, 83.4% (n = 131) samples were contaminated by coliform bacteria, of which 60.3% (n = 79) were from boreholes, while 39.7% (n = 52) were from wells.

### *Escherichia coli* confirmation

From the total samples contaminated by coliform (n= 131) bacteria detected, only 79 (60.3%) were confirmed to have *Escherichia coli* after performing the Eijkman Test. Of the contaminated water samples (n= 79) having *E. coli* detected in them, 52 representing 65.8% of the samples were from boreholes, while 27 representing 34.2% were from wells.

### Association between coliform and *Escherichia coli* against variables tested

The Table 1, Table 2 compared the association between different variables with detection of coliform and *E. coli* contaminants. The results of coliform detection determined that sampling site location (X^2^ value = 13.308, p value = 0.004), recent pump overhaul/repair (X^2^ value 13.308, p value = 0.003) and distance to pit latrine from water source (X^2^ value 9.113, p value = 0.021) had significant association. No association could be determined when detection of *E. coli* was compared to the variables tested.

**Table 1:** Comparing different variables against coliform contamination

Variables			Total	P. Value at 95% CI
	Coliform contamination			
**Location**	**Yes**	**No**		
Island	31	8	39	
Kisauni	26	4	30	**0.004**
Likoni	49	2	51	
Changamwe	25	12	37	
**Sample Source**				
Borehole	79	19	98	0.219
Wells	52	7	59	
**Protected Water Sources**				
Protected	119	25	144	0.369
Unprotected	12	1	13	
**Type of cover**				
Complete	119	25	144	
Partial	11	1	12	0.653
Open	1	0	1	
**Presence of Pump**				
Yes	120	25	145	0.425
No	11	1	12	
**Recent Overhaul**				
Yes	12	8	20	**0.003**
No	119	18	137	
Distance to water Source				
Between 1-10Metres	57	5	62	**0.021**
Equal to & Above 20 Metres	74	21	95	
Chlorine Treatment				
Yes	75	16	91	0.686
No	56	10		

**Table 2:** Comparing different variables against *escherichia coli* contamination

Variables			Total	P. Value at 95% CI
	*Escherichia coli*			
**Location**	**Yes**	**No**		
Island	20	19	39	
Kisauni	14	16	30	0.081
Likoni	32	19	51	
Changamwe	13	24	37	
Sample Source				
Borehole	52	46	98	0.376
Wells	52	7	59	
**Protected Water Sources**				
Protected	74	70	144	0.372
Unprotected	5	8	13	
Type of cover				
Complete	74	70	144	
Partial	4	8	12	0.295
Open	1	0	1	
**Presence of Pump**				
Yes	75	70	145	0.221
No	4	8	12	
**RecentOverhaul**				
Yes	6	14	20	0.052
No	73	64	137	
Distance to water Source				
Between 1-10Metres	37	25	62	0.058
Equal to & Above 20 Metres	42	53	95	
**Chlorine Treatment**				
Yes	45	46	91	0.798
No	34	32		

### Molecular characterization of *E. coli* pathotypes

**Figure 1:**
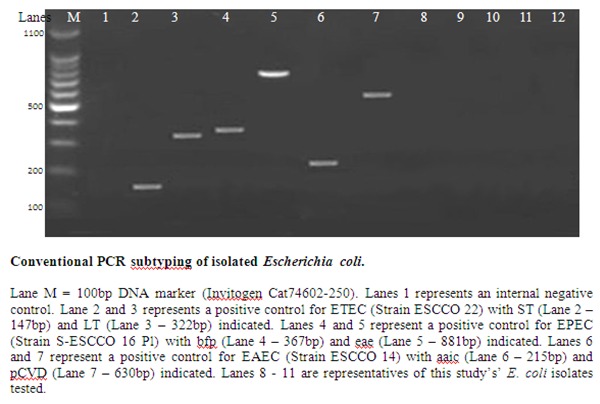
Representativeconventional PCR results of isolatedsamples

**Figure 2:**
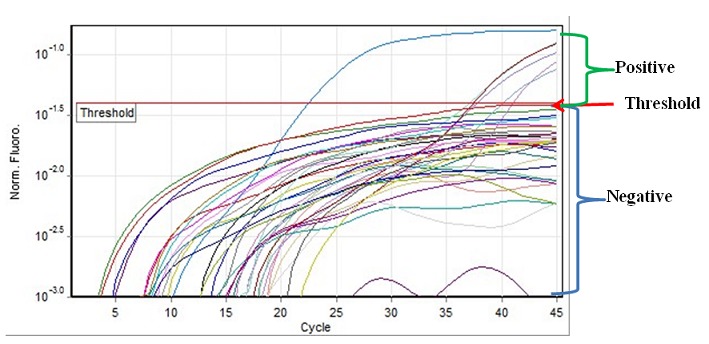
A representative Real time PCR results of molecular subtyping of isolated *E. coli*

To further characterize the *Escherichia coli* samples isolated in this study from the contaminated water samples, two molecular assays were carried out using type specific primers were used. The first test was a conventional multiplex PCR to detect three common pathotypes of *Escherichia coli* in developing countries that included ETEC, EPEC and EAEC. The primers (Table 3) eltB and estA target regions producing LT and ST enterotoxins in ETEC; while the primers eae and bfpA targeting EPEC; and the primers aaf targeting adhesion s in EAEC. The isolated E. coli were shown not to be of the three pathotypes (Figure 1). The second molecular test was a real time PCR that was carried out in order to detect all the six *E. coli* pathotypes including ETEC, EPEC, EAEC, EHEC, EIEC and DAEC. The isolated *E. coli* from this study were EIEC as shown in Figure 2 and representative real time PCR CT values (Table 4).

**Table 3:** Multiplex conventional PCR primers for ETEC, EPEC, and EAEC *E. Coli* strains

Primer sequence	Primer Name	Amplicon size (bp)
**LT-F**cacacggagctcctcagtc **LT-R**cccccagcctagcttagttt	**LT**	**508**
**ST-F**gctaaaccagtarggtcttcaaaa **ST-R**cccggtacargcaggattacaaca	**ST**	**147**
**bfpA-F**ggaagtcaaattcatggggg **bfpA-R**ggaatcagacgcagactggt	**bfpA**	**300**
**CVD-F**ctggcgaaagactgtatcat **CVD-R**caatgtatagaaatccgctgtt	**CVD432**	**650**
**aaiC-F**attgtcctcaggcatttcac **aaiC-R**acgacacccctgataaacaa	**aaiC**	**215**
**eae-F**cccgaattcggcacaagcataagc **eae-R**cccggatccgtctcgccagtattcg	**eae**	**881**

**Table 4:** CT Values of real time PCR

Color	Name	CT Value
Aqua	EIEC- 65	37.15
Light blue	EIEC- 70	40.09
Light purple	EIEC- 72	37.78
Purple	EIEC- 97	41.01
Dark red	EIEC- Positive Control	37.42
Blue	EIEC- Negative Control	

**Figure 3:**
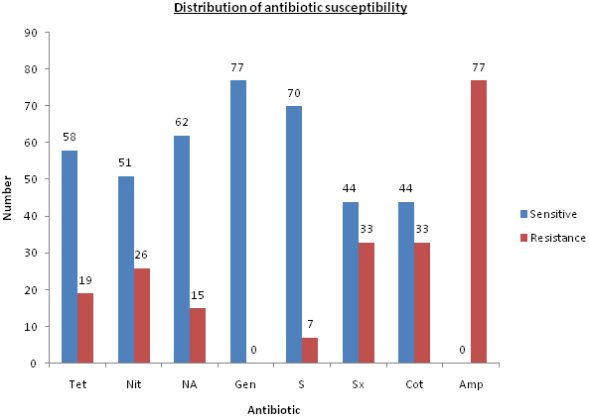
Bargraph indicating antibiotic susceptibility profile and distribution of isolated *E. coli*

**Antibiotic susceptibility profile of *E. coli* isolated** Antibiotic susceptibility profile of 77 *E. Coli* tested against 8 commonly used antibiotics in Mombasa County was carried out, where gentamicin (n= 77; 100%) was most sensitive, followed by streptomycin (n= 70; 90.9%). All the *E. coli* isolated were resistant to ampicillin (n= 77; 100%), followed by sulphamethoxazole (n= 33; 42.9%) and cotrimoxazole (n= 33; 42.9%). This is shown in Figure 3.

## Discussion

The development and standardization of bacteriological indicator organisms as indicators for faecal contamination of water has developed over time with coliform bacteria and *Escherichia coli* being finally accepted as indicators for faecal contamination of water samples [7, 8]. The presence of coliform bacteria and *E. coli* in water is an indicator of recent fecal contamination indicating the possible presence of disease-causing pathogens, such as bacteria, viruses, and parasites [9, 10]. This also led to the development of the standard, most probable number (MPN) that requires that less than one tube in all the dilutions used to detect contamination of water should show contamination. Anything above this value would indicate contamination of a water sample [7, 11]. Studies carried out in the same area show that there is the presence of both coliforms and *E. coli* [2]. This could be due to the lack of sanitation due to expansion of towns and or counties [2, 11]. The results of this study have indicated that majority of the samples collected by the study were contaminated by coliforms and also *E. coli* thus indicating recent faecal contamination. In this study, many variables were tested against coliform and *E. coli* detection, but only location of well samples collected, recent overhaul of sampling site and distance of water source and pit latrine indicated significant relationships when tested against the presence of coliforms but not *E. coli* detection. These similar significant relationships could not be established when compared to a previous study in the same area by Munga, and others in 2005. Studies in Zimbabwe have showed decreased contamination in distances that are more than 5 meters between the water source and source of contamination [12]. Even though this study did not look at practices of water storage, other previous studies have indicated that when people have collected water from protected water sources, they may have a tendency to consume the water without treatment because of belief chlorine will protect them [13], leading to outbreaks. Also other studies have showed that contamination of water sources can be linked to contaminated hands and collection containers such as cups [14]. Unprotected water sources have been implicated as sources of water contamination [14]. Also important to note is that, this study detected the *E. coli* subtype EIEC, which is associated with diarrhea [15]. This strain of *E. coli* causes watery, dysentery-like diarrhea, associated with cramps and fever [16]. It leads to the inflammation of the large intestines and occurs commonly in developing countries [16]. These bacteria in Kenya, have been isolated from stool samples [17], but have never been isolated from water sources before. A limitation to this study includes thenone detection of other bacterial species associated with water contamination from the samples. None determination of physical and chemical characteristics such as chlorine, calcium carbonate, ammonium, phosphates, nitrates and sulphates levels is also another limitation of this study is compared to other similar studies from the same County and in the country [2, 18]. Surveillance is an important tool in that it contributes to protection of health. According to WHO, in populations having more than 100,000 people, it is expected that the quality of drinking water samples should have proportions of between 85-99% clean/without *E. coli* [7].

## Conclusion

This current study's findings indicate high contamination of well and borehole water by coliforms and also specifically *E. coli* in Mombasa county. The *E. coli* isolates also varied patterns to commonly antibiotics used. Specifically, there was high resistance to ampicillin a commonly used antibiotic in Kenya. The *E. coli* isolates were very sensitive to gentamicin, streptomycin among other antibiotics. Continuous surveillance of faecal contamination of water should be carried out. Continuous health education to reduce contamination of water sources should be carried out in the county.

### What is known about this topic

- The detection of *E. coli* in water has been ongoing for a long time. This microorganism is associated with fresh fecal contamination of water sources. This is because the microorganism resides in the guts of animals, birds and humans and will only be found in water when open defecation occurs or when pit latrines are close to water sources such as boreholes and springs.

### What this study adds

- This study has gone further by not only detecting *E. coli* in portable water from boreholes and springs, but also subtyping the E. coli to determine which pathotypes are contaminating these water sources. This is the first time that this has been done in Kenya. Sub typing of *E. coli* is mainly done from diarrheal samples.
- The study has also looked at other factors distance between pit latrine and water sources and its effects on contamination of these sources. Again this study has highlighted that treatment of water with chlorine will not affect contamination with *E. coli* of these water sources. Probably the contamination is occurring from other sources.
